# The Potential Role of Electronegative High-Density Lipoprotein H5 Subfraction in RA-Related Atherosclerosis

**DOI:** 10.3390/ijms222111419

**Published:** 2021-10-22

**Authors:** Ching-Kun Chang, Wei-Chung Cheng, Wen-Lung Ma, Po-Ku Chen, Chu-Huang Chen, Pei-Chun Shen, Chia-Ching Chen, Shih-Hsin Chang, Yi-Hua Lai, Der-Yuan Chen

**Affiliations:** 1 Rheumatology and Immunology Center, China Medical University Hospital, Taichung 404, Taiwan; kun80445@gmail.com (C.-K.C.); pago99999@gmail.com (P.-K.C.); sherry61976@hotmail.com (S.-H.C.); T34404@mail.cmuh.org.tw (Y.-H.L.); 2 Translational Medicine Laboratory, China Medical University Hospital, Taichung 404, Taiwan; 3 The Ph.D. Program for Cancer Biology and Drug Discovery, China Medical University and Academia Sinica, Taichung 404, Taiwan; cwc0702@gmail.com; 4 Research Center for Cancer Biology, China Medical University, Taichung 404, Taiwan; y800418@gmail.com; 5 Graduate Institute of Biomedical Sciences, China Medical University, Taichung 404, Taiwan; maver-ick@mail.cmu.edu.tw; 6 Sex Hormone Research Center, Department of Obstetrics and Gynecology, China Medical University Hospital, Taichung 404, Taiwan; 7 Department of Nursing, Asia University, Taichung 413, Taiwan; 8 Vascular and Medicinal Research, Texas Heart Institute, Houston, TX 6770, USA; cchen@texasheart.org; 9 Institute for Biomedical Sciences, Shinshu University, Nagano 390-8621, Japan; 10 School of Medicine, Chang Gung University, Tao-Yuan 333, Taiwan; daminglife@gmail.com; 11 Ph.D. Program in Translational Medicine and Rong Hsing Research Center for Translational Medicine, National Chung Hsing University, Taichung 402, Taiwan; 12 College of Medicine, China Medical University, Taichung 404, Taiwan

**Keywords:** high-density lipoprotein (HDL), electronegative subfractions of HDL, H5, liquid chromatography/mass spectrometry (LC/MS), lipoprotein a (Lp(a)), atherosclerosis, rheumatoid arthritis (RA)

## Abstract

Although the heterogeneity of high-density lipoprotein-cholesterol (HDL-c) composition is associated with atherosclerotic cardiovascular risk, the link between electronegative subfractions of HDL-c and atherosclerosis in rheumatoid arthritis (RA) remains unknown. We examined the association of the percentage of the most electronegative subfraction of HDL-c (H5%) and RA-related atherosclerosis. Using anion-exchange purification/fast-protein liquid chromatography, we demonstrated significantly higher H5% in patients (median, 7.2%) than HC (2.8%, *p* < 0.005). Multivariable regression analysis revealed H5% as a significant predictor for subclinical atherosclerosis. We subsequently explored atherogenic role of H5 using cell-based assay. The results showed significantly higher levels of IL-1β and IL-8 mRNA in H5-treated (mean ± SD, 4.45 ± 1.22 folds, 6.02 ± 1.43-folds, respectively) than H1-treated monocytes (0.89 ± 0.18-folds, 1.03 ± 0.26-folds, respectively, both *p* < 0.001). In macrophages, H5 upregulated the mRNA and protein expression of IL-1β and IL-8 in a dose-dependent manner, and their expression levels were significantly higher than H1-treated macrophages (all *p* < 0.001). H5 induced more foam cell formation compared with H1-treated macrophages (*p* < 0.005). In addition, H5 has significantly lower cholesterol efflux capacity than H1 (*p* < 0.005). The results of nanoLC-MS/MS approach reveal that the best discriminator between high-H5% and normal-H5% is Apo(a), the main constituent of Lp(a). Moreover, Lp(a) level is a significant predictor for high-H5%. These observations suggest that H5 is involved in RA-related atherosclerosis.

## 1. Introduction

Atherosclerosis, a chronic inflammatory process with atheromatous plaque buildup in the arteries [1,2], is linked with increased atherosclerotic cardiovascular disease (ASCVD) risk. Rheumatoid arthritis (RA), a chronic inflammatory articular disease [3], is often complicated by premature or accelerated atherosclerosis and increased ASCVD risk [4,5]. ASCVD is even a major cause of mortality in RA, probably due to both conventional CVD risk factors and disease-associated systemic inflammation [5,6,7].

Regarding the link between high-density lipoprotein (HDL) cholesterol (HDL-c) and ASCVD, low HDL-c levels are conventionally associated with a high ASCVD risk [8]. HDL plays a crucial role in promoting cholesterol efflux and reversing cholesterol transport from the peripheral tissue to the liver. Other potential atheroprotective properties of HDL include the antioxidant, antiapoptotic, and anti-inflammatory effects. Camont et al. [9] used the liquid chromatography/mass spectrometry (LC/MS) approach to demonstrate the heterogeneity of HDL composition, which was probably reflected in the variations of its critical atherogenic properties. They also observed a significant correlation between the phosphosphingolipids components of HDL-c and the cholesterol efflux capability in human THP-1 macrophages. Small, dense, protein-rich HDL3 particles rich in negatively charged phospholipids have the antioxidative and anti-inflammatory properties.

Chronic inflammation may lead to the modifications of HDL-c compositions and thereby alter the protective functions of HDL-c [10]. The atherogenic HDL-c particles have recently been studied in a small-sample cohort of RA patients [11]. RA-associated inflammation may confer both quantitative and qualitative changes to HDL-c with the loss of some anti-inflammatory and atheroprotective properties [12]. Giraud et al. also revealed the change in the composition of HDL phosphosphingolipidome in RA patients [13]. These findings suggest that the heterogeneity of HDL-c composition and structure is linked to its functional alteration. However, the major determinants of the atherogenic function of the modified, proinflammatory HDL-c remain elusive, and it would be beneficial to identify the specific biomarkers for atherogenic HDL-c subfractions. To our best knowledge, there are no available data regarding the electronegative subfractions of HDL-c (H1-H5) or their atherogenic composition in RA-related atherosclerosis.

Lipoprotein a (Lp(a)), firstly detected by Berg in 1963 [14], is a macromolecular complex composed of Apolipoprotein(Apo) B-100 and Apo(a) [15]. Lp(a) has been reported as a biomarker capable of predicting ASCVD risk in RA [16], and a high Lp(a) level is related to the presence of carotid plaque [17]. Lp(a) levels were also negatively correlated with cholesterol efflux capacity of HDL-c [13] and positively associated with HDL-c levels [18]. However, Dursunoglu et al. revealed contradictory results that Lp(a) levels were negatively associated with HDL-c levels in RA [19].

In this pilot study, we aimed to investigate the association between the most electronegative subfraction of HDL (H5) and subclinical atherosclerosis in RA patients and examine the significantly different compositions of HDL-c between patients with high H5 percentage (H5%) and normal H5% by using the LC/MS approach. We also examined the effect of H5 on the expression of proinflammatory cytokine and cholesterol efflux capacity in vitro. Finally, we identified the potential predictors of H5% by using a linear regression analysis.

## 2. Results

### 2.1. Demographic Data and Clinical Characteristics of RA Patients

According to a previous report [20], a high H5% was defined as H5% ≥ 7.0%. Of the 69 RA patients, 38 (55.1%) had a high H5%, and 31 (44.9%) had normal H5% (Table 1). A trend of higher disease activity reflected by ESR, CRP, and DAS28 scores was observed in patients with high H5% compared to those with normal H5%, but there was no significant difference. No significant differences were observed in the demographic data, clinical characteristics, the proportion of medications use, or comorbidities between patients with high H5% and normal H5%. Moreover, there was no significant difference in the demographic data or body mass index among RA patients with high H5%, patients with normal H5%, and healthy subjects.

### 2.2. Comparison of Lipid Profiles, QRISK-2 Scores, and Subclinical Atherosclerosis between RA Patients with High H5% and Normal H5%

As illustrated in Table 1, RA patients with high H5% had a significantly higher prevalence of subclinical atherosclerosis shown by carotid sonography than those with normal H5%. However, there were no significant differences in plasma levels of lipid profile, atherogenic index, or QRISK-2 score between patients with high H5% and normal H5%. Although there was no statistical significance, the ccIMT was higher in RA patients with high H5% (1.28 mm, interquartile range (IQR) 1.16–1.40 mm) compared to those with normal H5% (1.14 mm, IQR 1.06–1.26 mm). The proportion of presence of carotid plaque was higher in RA patients with high H5% (28.9%) compared with those with normal H5% (16.1%), but there was no statistical significance.

### 2.3. Increased Plasma H5% in RA Patients

**Figure 1:**
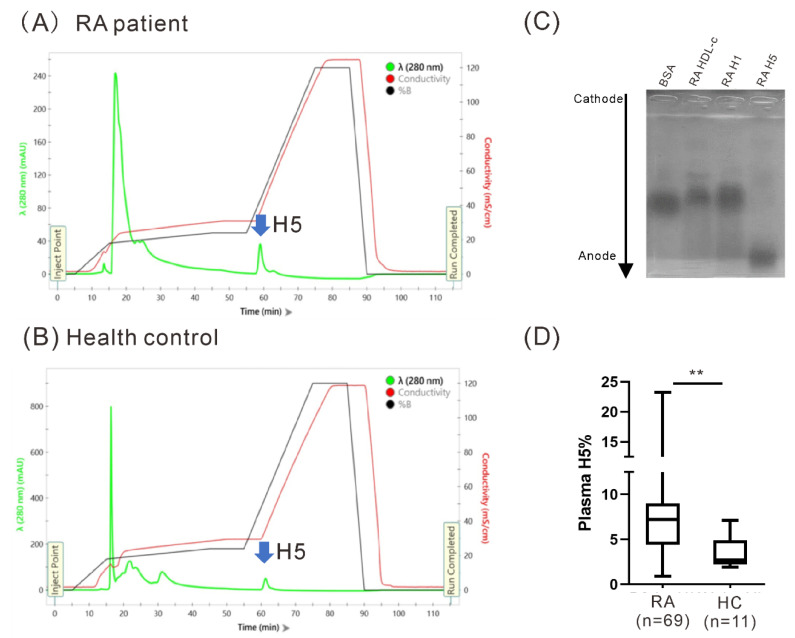
Analysis of HDL-c subfractions H5 from RA patients and HC individuals. According to electronegativity, HDL-c subfractions H1 and H5 were eluted at the indicated time points according to electronegativity using anion-exchange fast-protein liquid chromatography. Chromatograms are shown for (**A**) a RA patient and (**B**) healthy control. (**C**) HDL-c subfractions were subjected to agarose gel electrophoresis at 50 V for 2.4 h (BSA was used as a reference). (**D**) Comparisons of plasma H5% between RA patients and healthy controls. The data are presented as box-plot diagrams, in which the box encompasses the 25th percentile (lower bar) to the 75th percentile (upper bar). The horizontal line within the box indicates the median value for each group. BSA: Bovine serum albumin; HC: Healthy control; HDL-c: High-density lipoprotein cholesterol; RA: Rheumatoid arthritis. ** *p* < 0.005 vs. HC, determined using the nonparametric Mann–Whitney U test.

Representative distribution of the most electronegative subfraction of HDL-c (H5) (Figure 1A,B) in plasma samples from one RA patient and one healthy control, respectively, and electrophoretic mobility patterns for HDL-c subfractions H1 and H5 (Figure 1C). Plasma H5% were significantly higher in RA patients (median 7.2%, IQR 4.5–8.9%) than in healthy controls (median 2.8%, IQR 2.5–4.8%, *p* < 0.005; Figure 1D).

### 2.4. Logistic Regression Analyses and Receiver Operating Characteristic (ROC) Curve Analysis for Predicting the Presence of Subclinical Atherosclerosis

**Figure 2:**
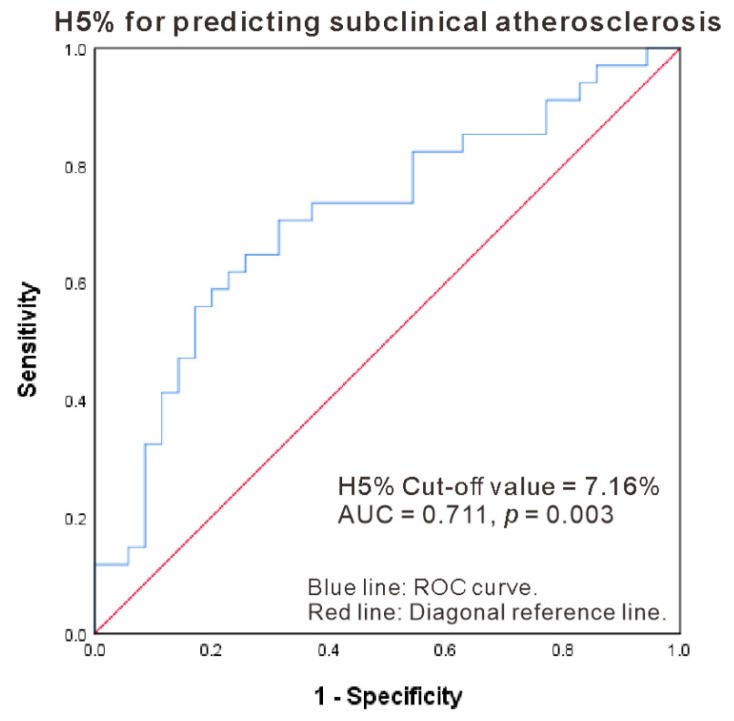
ROC curve analysis of plasma H5% for predicting the presence of subclinical atherosclerosis in RA patients. AUC: Area under ROC curve; Blue line: ROC curve; Red line: Diagonal reference line.

We further used logistic regression analysis to identify the potential markers including H5% for predicting the presence of subclinical atherosclerosis in RA patients. As illustrated in Table 2, a univariate regression analysis identified age and H5% as the potential predictors of subclinical atherosclerosis determined by ultrasound vascular imaging. The multivariable regression analysis demonstrated that H5% was a significant predictor of the presence of subclinical atherosclerosis after adjustment of age. Using ROC curve analysis, the optimal cut-off value of H5% for predicting the emergence of subclinical atherosclerosis was 7.16%, with an area under the ROC curve (AUC) of 0.711, the sensitivity of 70.6%, and specificity of 68.6% (*p* = 0.003, Figure 2).

### 2.5. The Effects of H5 on the Expression of Atherogenesis-Related Cytokine and Chemokine on THP-1 Monocytes or THP-1-Derived Macrophages

**Figure 3:**
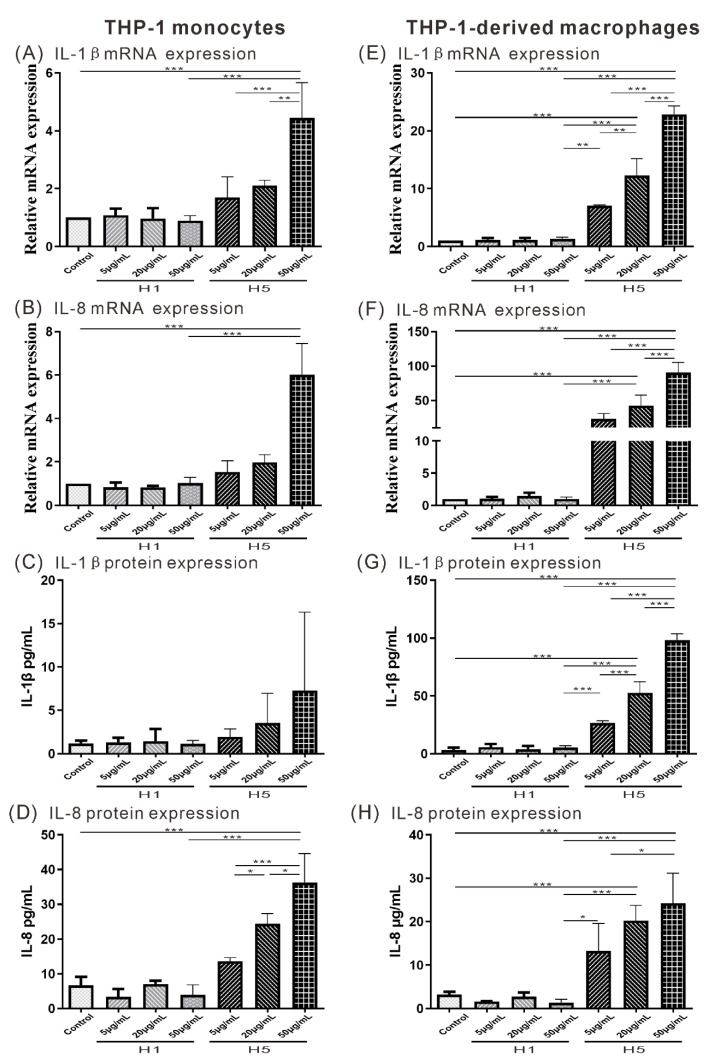
Effects of H5 on the expression of cytokines in monocytes and macrophages. The differences in the mRNA expression levels are shown for IL-1β (**A**,**E**), IL-8 (**B**,**F**) in THP-1 monocyte and THP-1-derived macrophage. The differences in the protein levels are shown for IL-1β (**C**,**G**), IL-8 (**D**,**H**) in THP-1 monocytes and THP-1-derived macrophages. * *p* < 0.05, ** *p* < 0.005, *** *p* < 0.001 determined by using one-way ANOVA test followed by Turkey’s post-test.

Given that H5% is a significant predictor of subclinical atherosclerosis in RA patients, we subsequently explored the potential roles of H5 in atherogenesis by using in vitro cell-based assay. Increasing evidence indicates that IL-1β and IL-8 are the key cytokine/chemokine in the atherogenesis [21,22,23,24]. We evaluated their expression levels of supernatants from H5- or H1-treated on THP-1 monocytes or THP-1-derived macrophages. The results showed that H5 upregulated the expression of IL-1β and IL-8 in both monocytes and macrophages (Figure 3A–H). The mRNA levels of IL-1β and IL-8 were significantly higher in monocytes treated with high-dose H5 (relative of control group, mean ± SD, 4.45 ± 1.22 folds, 6.02 ± 1.43 folds, respectively) than in those treated with high-dose H1 (0.89 ± 0.18 folds, 1.03 ± 0.26 folds, respectively, all *p* < 0.001, Figure 3A,B); IL-8 protein levels were also significantly higher in monocytes treated with high-dose H5 than in those treated with high-dose H1 or medium control (mean ± SD, H5: 36.29 ± 8.27 pg/mL, H1: 3.95 ± 2.89 pg/mL, control: 6.68 ± 1.43 pg/mL, all *p* < 0.001, Figure 3D), but no significant difference in IL-1β expression was observed (Figure 3C). In macrophages, H5 upregulated the expression of IL-1β and IL-8 in a dose-dependent manner. The mRNA levels of IL-1β and IL-8 were significantly higher in macrophages treated with 20 and 50 µg/mL H5 (relative of control group, mean ± SD, IL-1β, 20µg/mL 12.26 ± 2.95 folds, 50µg/mL 22.87 ± 1.41 folds; IL-8, 20µg/mL 42.32 ± 15.39 folds, 50µg/mL 90.70 ± 14.49 folds) than in those treated with high-dose H1 (IL-1β, 1.35 ± 0.26 folds; IL-8, 1.03 ± 0.25 folds, all *p* < 0.001, Figure 3E,F); the protein levels of IL-1β and IL-8 were also significantly higher in macrophages treated with 20 and 50 µg/mL H5 than in those treated with high-dose H1 or medium control (mean ± SD, IL-1β, 20µg/mL H5: 52.59 ± 9.54 pg/mL, 50µg/mL H5: 98.33 ± 5.38 pg/mL, H1: 5.35 ± 1.55 pg/mL, control: 3.29 ± 2.16 pg/mL; IL-8, 20µg/mL H5: 20.21 ± 3.56 µg/mL, 50µg/mL H5: 24.25 ± 6.90 µg/mL, H1: 1.38 ± 0.77 µg/mL, control: 3.20 ± 0.64 µg/mL, all *p* < 0.001, Figure 3G,H).

### 2.6. The Effects of H5 on Macrophage Foam Cell Formation

**Figure 4:**
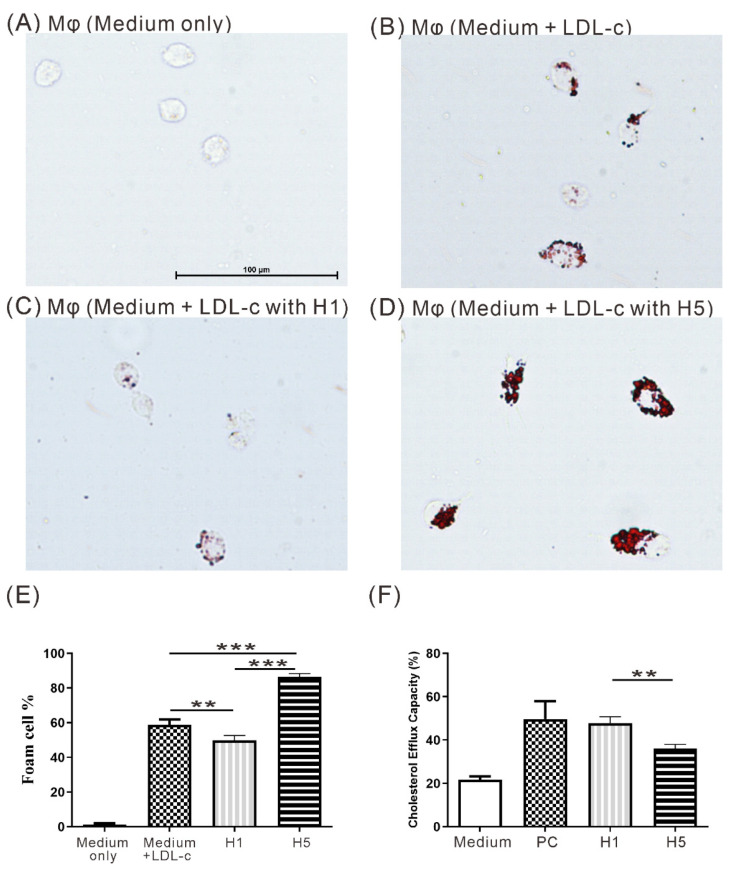
Effects of H5 on macrophage foam cell formation. THP-1-derived macrophages incubated with (**A**) culture medium only, (**B**) 50 μg/mL LDL, (**C**) 50 μg/mL LDL + 50 μg/mL H1, and (**D**) 50 μg/mL LDL + 50 μg/mL H5. (**E**) Difference in the proportion of macrophage foam cell formation among the different groups. ** *p* < 0.005, *** *p* < 0.001, determined by using one-way ANOVA test followed by Turkey’s post-test. (**F**) The cholesterol efflux capacity assay of H1 and H5. PC: Positive control (as kit reagent). ** *p* < 0.005, determined by using Student’s *t*-test.

To investigate the potential effects of electronegative H5 on macrophage foam cell formation, THP-1 cells were treated with PMA (10 nM) for 48 h to be differentiated into macrophage. Then the monocyte-derived macrophages were stimulated with 50 μg/mL LDL and subsequently treated with 50 μg/mL H1 or H5 for 48 h. As shown in Figure 4, the results revealed that the H5 induced more foam cell formation compared with LDL control or H1 treated macrophages (mean ± SD, 86.37 ± 2.03% in H5; 58.99 ± 2.88% in LDL control; 49.94 ± 2.65% in H1, all *p* < 0.005, Figure 4A–E).

### 2.7. The Effects of H5 on Cholesterol Efflux Capacity

Given that the HDL-c-induced cholesterol efflux from lipid-laden macrophages may prevent atherosclerosis [25], we evaluated the differences in cholesterol efflux capacity between H5 and H1. Our results showed that the cholesterol efflux capacity was significantly lower in H5-treated lipid-laden macrophages compared with H1-treated macrophages (mean 36.0% versus 47.9%, *p* = 0.004, Figure 4F).

### 2.8. Identification of Discriminative Compositional Proteins of HDL-c between RA Patients with High H5% and Normal H5%

**Figure 5:**
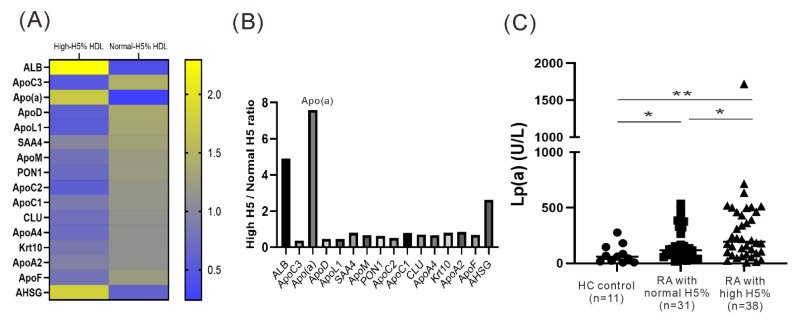
The H5 subfraction compositional analysis and verification. (**A**) The heatmap of high-H5% HDL and normal-H5% HDL using the nanoLC-MS/MS analysis. (**B**) The differentially expressed ratio of putative proteins between patients with high H5% and normal H5%. (**C**) Given Apo(a) is the main constituent of Lp(a), we examined the comparison of serum Lp(a) levels among patients with high H5%, patients with normal H5%, and healthy control (HC). * *p* < 0.05, ** *p* < 0.005, determined by using Mann–Whitney *U* test.

To further explore the difference in compositional proteins in HDL-c between patients with high H5% and normal H5%, we used the nanoLC-M/–MS analysis. Among the significantly differed compositional proteins, Apo(a) had the highest discriminative ability (7.58 folds, *p* < 0.001, Figure 5A,B) for detecting high H5% in RA patients.

### 2.9. Comparison of Lp(a) Levels among Patients with High H5%, Patients with Normal H5%, and HC

Because Apo(a) is the main constituent of Lp(a) [26], a marker of dysfunctional HDL [26,27], we evaluated the circulating Apo(a) levels, reflected by serum Lp(a) levels determined by ELISA. As shown in Figure 5C, significantly higher levels of Lp(a) were observed in RA patients with high H5% than in patients with normal H5% and in healthy subjects. Significantly higher Lp(a) levels were also observed in patients with normal H5% than in healthy subjects.

### 2.10. Linear Regression Analysis for H5%

Using a linear regression analysis with H5% as the dependent variable, the Lp(a) level closed to the set *p*-value (Table 3). The multivariable linear regression analysis also revealed the Lp(a) level was significantly associated with the H5%.

### 2.11. The Potential Role of H5 with RA-Related Atherogenesis in Clinical and In Vitro

**Figure 6:**
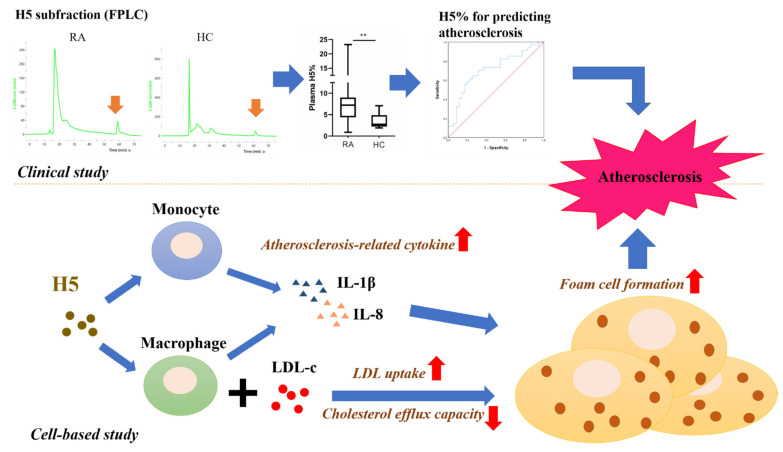
The potential role of H5 with RA-related atherogenesis in clinical and cell-based study. Fast protein liquid chromatography (FPLC) analysis shows higher H5% in RA than health control. The multivariable logistic regression analysis revealed H5% as a significant predictor of the presence of subclinical atherosclerosis. In cell-based experiments, H5 could promote the proinflammatory cytokine of IL-1β and IL-8, both cytokines significantly related to arteriosclerosis. Moreover, H5 has poor cholesterol efflux and promotes macrophages to uptake more LDL, leading to more foam cells formation, resulting in the occurrence of atherosclerosis. ** *p* < 0.005.

In this pilot study, we found that the percentage of the most electronegative subfraction of HDL-c (H5%) was significantly higher in RA patients than heath control. After adjusting for traditional risk factors, H5% was a significant predictor of subclinical atherosclerosis in RA patients. In cell-based experiments, we confirmed that H5 could upregulate the expression of IL-1β and IL-8, which were related to arteriosclerosis. Moreover, H5 has a poor cholesterol efflux and may promote macrophages uptake of more LDL, and finally increases the risk of atherosclerosis. The relevant results are also summarized as Figure 6.

## 3. Discussion

Increasing evidence indicates an association between the heterogeneity of HDL-c composition and ASCVD risk. Herein, we demonstrated for the first time that RA patients had significantly higher percentage of plasma H5, the most electronegative subfraction of HDL-c, than healthy controls. The multivariable logistic regression analysis revealed H5% as a significant predictor of the presence of subclinical atherosclerosis in RA. Furthermore, the in vitro cell-based assays showed that H5 could enhance proinflammatory cytokine expression and increased the foam cell formation. Similarly, the cholesterol efflux capacity assay revealed that H5 had significantly lower capacity of cholesterol efflux than H1. Among the compositional proteins in HDL-c, Apo(a) had the greatest individual ability to discriminate between patients with high H5% and normal H5%. As Apo(a) is the main constituent of Lp(a), our patients with high H5% had significantly higher Lp(a) levels than those with normal H5%. Serum Lp(a) levels are also probably a significant predictor of high H5% and the presence of subclinical atherosclerosis. These observations suggest that the H5 subfraction of HDL-c is linked to atherosclerosis in RA patients, and Lp(a) level may serve as a predictor of high H5% and atherogenic marker in this disease.

Although low HDL-c levels are generally associated with a high ASCVD risk [8], the subfraction of HDL-c that carries the most negative charge may contribute to atherogenicity in uremia patients [20]. Our RA patients, who have an increased risk of atherosclerosis [4,5], had elevated H5%, and patients with high H5% even had a significantly higher prevalence of subclinical atherosclerosis than those with normal H5%, indicating that H5% may be linked with RA-related atherosclerosis. Moreover, the multivariable logistic analysis showed H5% as a significant predictor of atherosclerosis. Using ROC analysis, we found that RA patients with plasma H5% above 7.16% have a high probability of subclinical atherosclerosis, with moderate sensitivity and specificity. The moderate sensitivity and specificity of H5% for predicting the emergence of subclinical atherosclerosis may be related to the small sample size of enrolled patients, the short duration of follow-up, and the probable influence of prescribed medications on lipid profile and H5% in this study. Therefore, our results require further confirmation by larger studies with a long-term follow-up period and enrollment of early RA patients. Besides, our results are preliminary and need further confirmation of their external validity.

As illustrated in the previous studies, HDL-c was conventional considered to be anti-inflammatory and negatively correlated with ASCVD risk [28,29]. Given a heterogeneity of HDL, the proinflammatory HDL-c has been reported to be associated with atherosclerotic plaque and metabolic abnormalities in RA [30,31,32]. These proinflammatory HDL also enhanced the levels of IL-1β and IL-8 [33,34,35]. IL-1β plays a key role in the inflammation pathway and modulates a lot of inflammatory components of atherosclerosis [22,36]. It also promotes macrophages to uptake LDL-c and then turn into foam cells [21]. Furthermore, targeting IL-1β may be a promising therapy strategy for ASCVD, as shown in clinical trials [37,38,39]. IL-8 levels were positively correlated to ASCVD risk and atherosclerosis [23,40,41]. Moreover, IL-8 had been reported to reduce cholesterol efflux of macrophage [42]. Using the cell-based assay to validate the functional role of HDL subfractions, we revealed a significant lower capacity of cholesterol efflux in H5-treated macrophages compared with H1-treated macrophages, consistent with the findings of a previous study [43]. These observations suggest that H5 is associated with atherosclerosis, perhaps through the upregulation of proinflammatory cytokine or chemokine and a reduction of cholesterol efflux capacity.

To identify the atherogenic composition of HDL-c in RA patients with high H5%, we examined the compositional characteristics of HDL-c by using proteomic analysis. Our patients with high H5% showed significantly higher Apo(a) levels, the main constituent of Lp(a) [26], than those with normal H5%. Lp(a) is increasingly recognized as a marker of dysfunctional HDL [26,27]. Vaisar et al. demonstrated that patients with coronary artery disease had significantly higher Lp(a) levels in HDL-c than healthy subjects [26]. Hippe et al. also revealed that elevated Lp(a) levels were an independent predictor of increased ASCVD burden in Caucasians [44]. A large-scale study in the Chinese population also showed that Lp(a) might be a biomarker for ASCVD [45]. In the present study, we revealed significantly higher Lp(a) levels in RA patients with high H5% compared to those with normal H5% and healthy subjects. Given the close link between Lp(a) and ASCVD, high H5% is probably involved in RA-related atherosclerosis.

Despite the novel findings in this pilot study, there were still some limitations. First, the sample size of RA patients (n = 69) was relatively small, and the duration of follow-up was short (2 years), which may reduce the statistical power. Although there was no significant difference in the proportion of conventional synthetic disease-modifying antirheumatic drug (csDMARDs) use between patients with high H5% and normal H5%, the possible effects of csDMARDs on plasma H5% remain to be clarified. Although previous studies have revealed the effects of statins on plasma HDL-c [46], we did not evaluate the changes of plasma HDL-c levels or H5% after the use of statins. Besides, the number of samples executed in the LC-MS/MS analysis may not be sufficient enough to detect all the compositional proteins in HDL-c from our patient with high H5%. A long-term study of a larger group of RA patients is required to confirm our findings.

## 4. Materials and Methods

### 4.1. Study Population

Sixty-nine patients who met the 2010 revised criteria of the American College of Rheumatology for RA [47] in active status were enrolled. Disease activity was assessed by using the 28-joint disease activity score (DAS28) [48], and active status was defined as DAS28 ≥ 3.2. Each patient had previously received corticosteroids, nonsteroidal anti-inflammatory drugs, and at least one of csDMARDs. Patients with a recent history (i.e., within one year before enrollment) of coronary heart disease or ischemic stroke were excluded. Follow-ups for the emergence of ASCVD, which included acute myocardial infarction and ischemic stroke, had been done for at least two years. Eleven sex- and age-matched healthy volunteers who had no rheumatic disease were enrolled as healthy controls (HC). The Institutional Review Board of our hospital approved this study (CMUH107-REC2-038 and CMUH109-REC3-161), and each participant’s written consent was obtained according to the Declaration of Helsinki.

### 4.2. Cell Culture

The human monocytic cell line, THP-1 cells (TIB-202, American Type Culture Collection, Manassas, VA, USA), was grown in RPMI 1640 medium (Thermo Fisher Scientific, Waltham, MA, USA) supplemented with 10% FBS (Thermo Fisher Scientific, Waltham, MA, USA) and 1% penicillin/streptomycin antibiotics in an incubator (Thermo Fisher Scientific, Waltham, MA, USA) containing 5% CO_2_ at 37 °C.

### 4.3. Determination of Plasma Lipid Profiles and Atherogenic Index (AI)

All the blood samples were collected from the participants in the early morning after an overnight fast for 12 h. Plasma levels of total cholesterol (TC), triglyceride, HDL-c, and low-density lipoprotein (LDL) cholesterol (LDL-c) were measured using enzymatic methods with a chemistry analyzer AU5800 (Beckman Coulter, California, CA, USA) according to the manufacturer’s instructions. The AI was calculated as the ratio of TC/HDL-c.

### 4.4. Measurement of 10-Year Risk of Cardiovascular Disease (QRISK-2 Score)

The global 10-year risk for a heart attack or ischemic stroke was estimated by calculating the QRISK-2 scores on the following website (https://www.qrisk.org assessed on 22 February 2021) [49]. Briefly, factors including age, sex, ethnicity, physical characteristics, total cholesterol/HDL-c ratio, self-reported smoking status, diabetic status, the presence of kidney disease, and family history of heart disease were considered to determine the QRISK-2 score for each study participant.

### 4.5. Isolation and Fractionation of HDL-c

HDL-c isolation with sequential potassium bromide (KBr) density ultracentrifugation as described [50]. To the plasma was added 1% antibiotics (penicillin/streptomycin stock solution, Thermo Fisher Scientific, Waltham, MA, USA), 0.5 mM EDTA (Thermo Fisher Scientific, Waltham, MA, USA), protease inhibitor cocktail (cOmplete^TM^, Roche, Basel, Switzerland) to avoid ex vivo oxidation and degradation. Purified HDL-C was dialyzed against a degassed solution of Buffer A (20 mM Tris-HCl plus 0.5 mM EDTA) on a shaker at 4 °C with five buffer changes (once/day).

### 4.6. Isolation of HDL-c Subfractions with Anion-Exchange Column

Dialyzed HDL-c were separated to different subfractions by using UnoQ6 anion-exchange columns (Bio-Rad, Hercules, CA, USA) with an NGC Quest 10 chromatography system (Bio-Rad, Hercules, CA, USA) as described [43]. HDL-c in 3 mL was injected onto a UnoQ6 column and eluted with a multistep gradient of buffer B (1M NaCl in buffer A) at 2 mL/min rate. Five HDL-c subfractions were eluted with a multistep gradient of buffer B according to electronegativity. H1 was the effluent collected between fractions 8 to 11 (14–22 min) and H5 fractions 30 to 35 (58–70 min). The respective fractions were then concentrated with Vivaspin Turbo (Sartorius, Göttingen, Germany) and sterilized by passage through 0.22 μm syringe filters (Fintech, Changhua, Taiwan).

### 4.7. Agarose Gel Electrophoresis of HDL-c Subfractions

To confirm the different electric charges on the separated HDL-c subfractions, we loaded 5 µg of each subfraction onto a 0.72% agarose gel (in Tris/Borate/EDTA buffer) and used the negatively charged protein bovine serum albumin (BSA) as a marker. Gel electrophoresis was performed at 50 V for 2.4 h, followed by staining with Simply Blue SafeStain (Thermo Scientific, Waltham, MA, USA).

### 4.8. IL-1β and IL-8 mRNA Expression in THP-1 Cells Treated with H1 or H5

The human monocytic cell line, THP-1 cells (BCRC 60430; Bioresource Collection and Research Center, Hsinchu, Taiwan), was grown in RPMI 1640 (Thermo Fisher Scientific, Taichung, Taiwan) supplemented with 10% FBS and 1% penicillin/streptomycin antibiotics in an incubator (Thermo Fisher Scientific GmbH, Dreieich, Germany) containing 5% CO_2_ at 37 °C. To induce differentiation into macrophages, THP-1 cells were grown in media and treated with 10 ng/mL phorbol myristate acetate (MilliporeSigma, Temecula, CA, USA) for 48 h. Then cells were treated with H1 or H5 (5, 20, 50 μg/mL) at 37 °C for 2 days. The expression levels of each gene were determined by the CFX96 Real-time PCR system (BioRad, Hercules, CA, USA) with IQ SYBR Green Supermix reagent (BioRad). The primer sequences are as follows: IL-1β, 5′-ACAGA-TGAAG-TGCTC-CTTCC-A-3′ (forward) and 5′-GTCGG-AGATT-CGTAG-CTGGA-T-3′ (reverse); IL-8, 5′-GAGAG-TGATT-GAGAG-TGGAC-CAC-3′ (forward) and 5′-CACAA-CCCTC-TGCAC-CCAGT-TT-3′ (reverse); and Actin, 5′-CAAGA-TCATT-GCTCC-TCCTG-3′ (forward) and 5′-ATCCA-CATCT-GCTGG-AAGG-3′ (reverse). The difference in expression in the target gene relative to the averaged internal control gene was calculated by 2^−^^ΔCt^, ΔCt = Ct_target gene_ − Ct_Actin_.

### 4.9. Enzyme-linked Immunosorbent Assay (ELISA)

The cultured supernatant from monocyte- and macrophage-treated H1/ H5 was harvested, and we performed ELISA. The cytokines level was determined by IL-1β ELISA Kit (BioLegend, San Diego, CA, USA) and IL-8 ELISA Kit (BD Biosciences, San Diego, CA, USA).

Serum levels of Lp(a) were determined using ELISA according to the manufacturer’s instruction (Mercodia, Uppsala, Sweden). The optical density at 450 nm was determined using a microplate absorbance reader Infinite M1000 Pro (TECAN, Männedorf, Switzerland). The data were analyzed with four parameters logistic curve on Myassays web site (https://www.myassays.com/ assessed on 8 March 2021). The unit of Lp(a) was presented as U/L; one U/L is equal to 1.254 mg/L.

### 4.10. Examination of Foam Cells Formation in THP-1-Derived Macrophages

THP-1 cells were grown in media and treated with 10 ng/mL phorbol myristate acetate (MilliporeSigma, Temecula, CA, USA) to induce differentiation into macrophage for 48 h. The culture medium was subsequently changed to RPMI and 1% lipid-depleted fetal bovine serum (FBS) from differential ultracentrifugation, as described previously [51]. Then, the macrophages were treated 50 μg/mL LDL with 50 μg/mL H1 or H5 at 37 °C for 48 h. Foam cell formation in macrophages was examined by using Oil red O staining [52]. The images for foam cell formation were observed in an ECLIPSE 50i microscope (Nikon, Tokyo, Japan) and ECHO Retrieval system (ECHO, San Diego, CA, USA). The percentage of foam cell formation was quantitated by dividing the number of Oil red O staining macrophages by the total number of macrophages in 3 random microscopic fields (over 200 cells).

### 4.11. Cholesterol Efflux Capacity Assay

The cell-based cholesterol efflux capacity assay was performed according to the manufacturer’s instruction (ab196985, Abcam, Cambridge, UK). In detail, THP-1 cells were plating in 96-well plate and added 100 nM Phorbol myristate acetate (Sigma, Temecula, CA, USA) in culture medium for 48 h. We washed the cell monolayer with RPMI 1640 medium (without serum). Then, we premixed Labeling Reagent and Equilibration Buffer (1:1) and added 100 µL per well. We incubated the plate overnight (16 h) protected from light in cell incubator. Then, we removed the culture medium and washed by no serum RPMI 1640 medium. Then, the cells were treated with 50 µg H1 or H5 at 37 °C for 4 h. We transferred the supernatant to a 96-well plate and added 100 µL cell Lysis Buffer to the original plate and shook on plate shaker for 30 min. We measured the fluorescence (Ex/Em = 482/515 nm) with the microplate absorbance reader Infinite M1000 Pro (TECAN, Männedorf, Switzerland). The cholesterol efflux rate was calculated by (Fluorescence of medium)/(Fluorescence of medium + cell lysate).

### 4.12. NanoLC-MS/MS Analysis of HDL-c Composition

The HDL-c proteome analysis was performed using the nanoLC-MS/MS by T-BAL (Taiwan-BioActive Lipid, Taichung, Taiwan), as described previously [53]. Briefly, HDL-c was added to cold acetone (4-folds volume) at −20 °C overnight, centrifuged at 12,000× *g* at 4 °C for 10 min, and then the supernatant was removed. The protein pellet was dissolved in 8M urea/2M thiourea solution. NanoLC-MS/MS was performed with a nanoflow ultraperformance liquid chromatography system (Ulti-Mate 3000 RSLCnano system, Thermo Scientific, Waltham, MA, USA) coupled to a hybrid quadrupole time-of-flight (Q-TOF) mass spectrometer (maXis Impact, Billerica, Bruker). After sample loading, the peptides were eluted from a trap column into an analytical column (Acclaim PepMap C18, Thermo Scientific, Waltham, MA, USA) coupled to a nanoelectrospray ionization source on the Q-TOF mass spectrometer. The nanoLC gradient conditions were as follows: 5% to 45% (*v*/*v*) buffer B (80%ACN/0.1% FA) for 55 min, and then to 99% B for 5 min; hold at 99% B for 5 min, then return to 95% buffer A (2%ACN/0.1% FA) for 9 min. Seven precursors of charge +2, +3, and +4 from each TOF MS scan were dynamically selected and isolated for MS/MS fragment ion scanning. The selected precursors were then actively excluded for 15 s. The MS and MS/MS accumulation were set at 2 and 10 Hz, respectively.

### 4.13. Protein Database Search and Label-Free Quantification

The spectra acquired by nanoLC-MS/MS were converted into xml files using DataAnalysis (version 4.1, Billerica, Bruker, MA, USA), as described previously [54]. For each spectrum, PEAKS software (PEAKS^®^ studio X, Bioinformatics Solutions, Waterloo, ON, Canada) was used to output a confidence level of amino acid sequences based on de novo sequencing. UniProt database was used for protein identification. The search parameters for precursor ion and fragment ion tolerance were 50 ppm and 0.05 Da, respectively. The following search parameters were selected: Taxonomy, Human; missed cleavages, 2; enzyme, trypsin; fixed modifications, carbamidomethyl(C); and variable modifications, oxidation (M) and deamidation (NQ). False discovery rates (FDRs) were estimated to be < 1%. Data-dependent acquisition (DDA) was performed for protein quantification. The mass error tolerance was 50 ppm, and the retention time shift tolerance was 1 min. After identification, the label-free function of PEAKs was used to obtain the relative abundance of proteins among the groups.

### 4.14. Ultrasound Vascular Imaging of Carotid Arteries

Ultrasound vascular imaging of the carotid arteries included the measurement of ccIMT and the detection of focal plaque in the extracranial carotid tree. Carotid plaque was defined as a localized thickening > 1.5 mm that did not uniformly involve the whole artery. A ccIMT > 1.2 mm or the presence of carotid plaque is used to define subclinical atherosclerosis with a minor modification of previous reports [55,56].

### 4.15. Statistical Analysis

The results are presented as the mean ± standard deviation (SD), the standard error of the mean (SEM), or the median (interquartile range). The nonparametric Mann–Whitney U test was used for between-group comparisons of numerical variables. The one-way ANOVA test followed by Turkey’s post-test was used for multiple groups. We also constructed a univariate and multiple logistic regression model to evaluate factors contributing to the presence of subclinical atherosclerosis detected by ultrasonography, including traditional risk factors such as age, gender, smoking, and hypertension. The optimal cut-off values of HDL-H5 proportion (H5%) for predicting the presence of subclinical atherosclerosis in RA patients were determined by ROC curve analysis. We also constructed a univariate and multiple linear regression model to evaluate with H5%. A two-sided *p*-value < 0.05 was considered statistically significant. All the plots and statistical analysis were performed using IBM SPSS Statistics v25 (IBM, New York, NY, USA) and GraphPad Prism v9.1 (GraphPad Software, San Diego, CA, USA).

## 5. Conclusions

This pilot study reveals that H5% is elevated in RA patients and high H5% is a significant predictor of subclinical atherosclerosis. The results of in vitro cell-based assays that H5 upregulate the expression of atherogenic IL-1β/IL-8 and enhance the foam cell formation suggest that H5 may contribute to RA-associated atherosclerosis. The proteomic analysis reveals that the best discriminator between high-H5% and normal-H5% is Apo(a), the main constituent of Lp(a). The Lp(a) level is a significant predictor for high H5%. These observations indicate that H5 is involved in RA-related atherosclerosis. Long-term follow-ups are needed to evaluate the predictive value of plasma H5% for ASCVD risk in RA patients. Although these results are preliminary, our findings may provide new insight into the electronegative heterogeneity of HDL-c in RA patients. In clinical practice, plasma H5% evaluation would be helpful to identify RA patients at risk of developing subclinical atherosclerosis, for whom close surveillance for the emergence of ASCVD is necessary.

## Figures and Tables

**Table 1:** Demographic and laboratory data in rheumatoid arthritis (RA) patients with a high percentage of H5 in high-density lipoprotein cholesterol (HDL-c) (H5%), RA patients with normal H5%, and healthy controls ^a^.

	RA with High H5% (*n* = 38)	RA with Normal H5% (*n* = 31)	Healthy Controls (*n* = 11)
Age at study entry, years	59.6 ± 12.4	55.5 ± 13.6	40.9 ± 9.9
Women proportion	29 (76.3%)	23 (74.2%)	7 (64%)
RA duration, months	70.5 ± 29.1	70.7 ± 26.5	NA
BMI, kg/m^2^	24.5 ± 4.4	23.0 ± 2.3	23.3 ± 2.3
RF positivity	28 (73.7%)	22 (71.0%)	NA
ACPA positivity	27 (71.1%)	20 (64.5%)	NA
ESR at study entry, mm/1st hour	32.5 ± 25.1	23.0 ± 17.5	NA
CRP at study entry, mg/dl	2.80 ± 5.03	1.61 ± 2.47	NA
DAS28 at study entry	4.66 ± 1.72	4.18 ± 1.73	NA
csDMARDs alone at study entry	16 (42.1%)	14 (45.2%)	NA
Biologics used at study entry			
Tofacitinib	10 (26.3%)	8 (25.8%)	NA
TNF-α inhibitors	6 (15.8%)	5 (16.1%)	NA
IL-6R inhibitor	5 (13.2%)	3 (9.7%)	NA
Abatacept	1 (2.6%)	1 (3.2%)	NA
Comorbidities			
Hypertension	23 (60.5%)	17 (54.8%)	0 (0.0%)
Diabetes mellitus	3 (7.9%)	4 (12.9%)	0 (0.0%)
Current smoker	3 (7.9%)	3 (9.7%)	0 (0.0%)
Coronary artery disease	3 (7.9%)	2 (6.5%)	0 (0.0%)
Chronic kidney disease	3 (7.9%)	1 (3.2%)	0 (0.0%)
Lipid profile at study entry			
TC, mg/dL	192 (166–215)	196 (158–227)	172 (146–215)
HDL-c, mg/dL	57 (47–66)	54 (44–69)	41 (43–59)
Triglyceride, mg/dL	89 (53–116)	102 (74–135)	73 (33–122)
LDL-c, mg/dL	111 (95–124)	124 (85–146)	111 (92–141)
Atherogenic index	3.3 (2.7–4.1)	3.3 (3.0–4.3)	3.1 (2.8–4.2)
QRISK-2 score, %	6.9 (3.5–13.7)	4.5 (2.5–13.6)	0.4 (0.3–1.0)
ccIMT, mm	1.28 (1.16–1.40)	1.14 (1.06–1.26)	NA
Carotid plaque	11 (28.9%)	5 (16.1%)	NA
Subclinical atherosclerosis	25 (65.8%) ^b^	9 (29.0%)	NA

^a^ Data are presented as the median (interquartile range, IQR), mean ± SD, or number (percentage). ^b^ *p* < 0.005, vs. RA patients with normal H5%, as determined by using the Mann–Whitney U test. BMI: Body mass index; RF: Rheumatoid factor; ACPA: Anticitrullinated peptide antibodies; ESR: Erythrocyte sedimentation rate; CRP: C-reactive protein; DAS28: Disease activity score for 28 joints; csDMARDs: Conventional synthetic disease-modifying antirheumatic drugs; TNF-α: Tumor necrosis factor-α; IL-6: Interleukin-6; TC: Total cholesterol; HDL-c: High-density lipoprotein cholesterol; LDL-c: Low-density lipoprotein cholesterol; ccIMT: Common carotid artery intima-media thickness; NA: Not applicable. Carotid plaque was defined as a localized thickening > 1.5mm that did not uniformly involve the whole artery. The presence of subclinical atherosclerosis was determined by using carotid ultrasonography.

**Table 2:** Logistic regression analysis of traditional cardiovascular risk factors, RA-related factors, and plasma H5% with the presence of subclinical atherosclerosis in 69 patients with RA.

Risk Factor	*p*-Value	Odds Ratio	95% Confidence Interval
Univariate			
Gender (Female)	0.367	0.6	0.198–1.819
Age	0.003	1.081	1.028–1.138
TC	0.717	0.998	0.987–1.009
TG	0.537	1.002	0.995–1.009
HDL-c	0.331	0.983	0.95–1.017
LDL-c	0.879	0.999	0.987–1.012
TC/HDL-c	0.986	1.003	0.689–1.461
BMI	0.572	0.966	0.858–1.088
DM	0.97	1.032	0.193–5.51
AF	0.976	1.031	0.137–7.769
H5%	0.007	1.265	1.066–1.501
Smoking	0.687	1.336	0.327–5.467
Hypertension	0.888	1.071	0.412–2.788
Multivariable			
Age	0.005	1.077	1.023–1.134
H5%	0.014	1.255	1.047–1.506

RA: Rheumatoid arthritis; TC: Total cholesterol; TG: Triglyceride; HDL-c: High-density lipoprotein cholesterol; LDL-c: Low-density lipoprotein cholesterol; BMI: Body mass index; DM: Diabetes mellitus; AF: Atrial fibrillation; H5%: The percentage of the most electronegative subfraction of HDL-c.

**Table 3:** Linear regression of baseline levels of lipid profile, RA-related inflammatory parameters, and Lp(a) levels for predicting H5% in 69 patients with RA.

Factor	*p*-Value	β-Value	95% Confidence Interval
Univariate			
Age	0.259	0.138	−0.104–0.379
Gender (Female)	0.439	−0.095	−0.337–0.148
LDL-c	0.452	−0.095	−0.346–0.156
HDL-c	0.865	−0.021	−0.275–0.231
TC/HDL-c	0.712	−0.047	−0.304–0.208
DAS28	0.678	−0.051	−0.294–0.193
ESR	0.917	0.013	−0.231–0.257
CRP	0.894	−0.016	−0.260–0.227
Lp(a)	0.064	0.225	−0.013–0.462
Multivariable			
Age	0.264	0.175	−0.138–0.493
Gender (Female)	0.685	−0.058	−0.337–0.223
LDL-c	0.249	−0.311	−0.852–0.226
HDL-c	0.880	−0.037	−0.528–0.454
TC/HDL-c	0.727	0.123	−0.576–0.821
DAS28	0.944	−0.020	−0.607–0.565
ESR	0.538	−0.210	−0.960–0.507
CRP	0.982	−0.004	−0.368–0.360
Lp(a)	<0.05 (0.028)	0.299	0.032–0.551

RA: Rheumatoid arthritis; LDL-c: Low-density lipoprotein cholesterol; HDL-c: High-density lipoprotein cholesterol; TC: Total cholesterol; TG: Triglyceride; DAS28: Disease activity score for 28 joints; ESR: Erythrocyte sedimentation rate; CRP: C-reactive protein.

## Data Availability

The data that support the findings of this study are available from the corresponding author (D.-Y.C.) upon reasonable request.

## Abbreviations

ACPA	Anticitrullinated peptide antibodies
CRP	C-reactive protein
AF	Atrial fibrillation
AI	Atherogenic index
Apo	Apolipoprotein
ASCVD	Atherosclerotic cardiovascular disease
BMI	Body mass index
BSA	Bovine serum albumin
csDMARD	Conventional synthetic disease-modifying antirheumatic drug
DAS28	Disease activity score for 28 joints
DM	Diabetes mellitus
ELISA	Enzyme-Linked Immunosorbent Assay
ESR	Erythrocyte sedimentation rate
FPLC	Fast protein liquid chromatography
H5%	The percentage of the most electronegative subfraction of HDL-c
HC	Healthy controls
HDL	High-density lipoprotein
HDL-c	High-density lipoprotein cholesterol
IL	Interleukin
JAKi	Janus kinase inhibitors
LC/MS	Liquid chromatography/mass spectrometry
LDL	Low-density lipoprotein
LDL-c	Low-density lipoprotein cholesterol
Lp(a)	Lipoprotein a
RA	Rheumatoid arthritis
RF	Rheumatoid factor
TC	Total cholesterol
TCZ	Tocilizumab
TG	Triglyceride
TNF-α	Tumor necrosis factor-α
